# Predict the role of lncRNA in kidney aging based on RNA sequencing

**DOI:** 10.1186/s12864-022-08479-8

**Published:** 2022-04-02

**Authors:** Jie Li, Fanfan Gao, Limin Wei, Lei Chen, Ning Qu, Lu Zeng, Yulong Luo, Xinmei Huang, Hongli Jiang

**Affiliations:** 1 Dialysis Department of Nephrology Hospital, The First Affiliated Hospital of Xi’an Jiaotong University, West Yanta Road 277, 710061 Xi’an, Shaanxi China; 2 Department of Nephrology, The First People’s Hospital Lanzhou City, No1 Wujiayuan, 730050 Qilihe, Lanzhou, Gansu China

**Keywords:** lncRNA, Kidney, Aging

## Abstract

**Background:**

Long noncoding RNAs (lncRNAs) are involved in physiological and pathological processes. However, no studies have been conducted on the relationship between lncRNAs and renal aging.

**Results:**

First, we evaluated the histopathology of young (3-month-old) and old (24-month-old) C57BL/6J mouse kidneys. Masson trichrome staining and PAS staining showed interstitial collagen deposition and fibrosis, mesangial matrix expansion, a thicker basement membrane and renal interstitial fibrosis in old mouse kidneys. Senescence-associated β-galactosidase (SA-β-gal)-positive areas in the kidneys of old mice were significantly elevated compared to those of young mice. Then, we analyzed the differential expression of lncRNAs and mRNAs in the kidneys of young and old mouse kidneys by RNA-seq analysis. 42 known and 179 novel differentially expressed lncRNAs and 702 differential mRNAs were detected in the mouse kidney. Next, we focused on the differentially expressed mRNAs and lncRNAs by RNA-seq. GO and KEGG analyses were performed based on differentially expressed mRNAs between young and old mouse kidneys. Transregulation based on RIsearch and the correlation coefficient of mRNA-lncRNA were also calculated. The mRNA-lncRNA network was constructed by choosing a Spearman correlation coefficient > 0.9 or <-0.9. GO and KEGG pathway enrichment analyses revealed that differentially expressed mRNAs participated in aging-related pathways. A total of 10 lncRNAs and *trans*-regulated mRNAs were constructed. Finally, we validated the role of lncRNA *Gm43360* by CCK-8, flow cytometry, western blot and SA-β-gal staining. The expression level of *Adra1a* was positively correlated and *Csnk1a1* was negatively correlated with lncRNA *Gm43360*. The cell counting kit-8 (CCK-8) results showed that lncRNA *Gm43360* promoted cell viability. LncRNA *Gm43360* increased the percentage of S phase cells and decreased the percentage of G1 phase cells compared with the negative control. LncRNA *Gm43360* decreased the expression of p53, p21 and SA-β-gal.

**Conclusions:**

LncRNA *Gm43360* may play a protective role in kidney aging.

**Supplementary Information:**

The online version contains supplementary material available at 10.1186/s12864-022-08479-8.

## Background

According to statistics from the United Nations, the average human life expectancy has doubled in most developed countries over the last 200 years; therefore, the question of how to improve the quality of life of the elderly has become a major public health problem [1, 2]. Aging is a continuous and gradual process and has been generally identified as the starting site for several chronic diseases, including cardiovascular system disease [3], metabolic system disorder [4], cancer [5, 6], and neurodegenerative disease [7]. The glomerular filtration rate (GFR) declines with aging, the kidneys process metabolic toxins at a slower rate, and the accumulation of metabolic toxins accelerates aging [8, 9]. Exploration of the mechanism of kidney aging will be of great significance for delaying the occurrence and development of renal aging. Although a small number of studies have been conducted on renal aging, it is still meaningful to comprehend the mechanism of renal aging.

Long chain noncoding RNAs (lncRNAs) are more than 200 nucleotides in length. LncRNAs regulate transcriptional and posttranscriptional RNA processing, translation, DNA methylation, and chromatin architecture via local (*cis*) and distal (*trans*) mechanisms. It is generally considered that they do not encode proteins but can be involved in various biological regulatory functions. Lin suggested that the lncRNA *HOTAIR* promotes the development of Parkinson’s disease by targeting miR-126 [10]. Gu suggested that the lncRNA *EBF3-AS* promotes neuronal apoptosis in Alzheimer’s disease [11]. J-X Pan suggested that lncRNA *H19* promotes atherosclerosis progression. LncRNA *H19* was recently reported to play a crucial role in the activation of MAPK and the NF-kB signaling pathway and the induction of atherosclerosis [3]. lncRNAs play crucial roles in the progression of diabetic nephropathy [12], glomerular disease [13] and renal fibrosis [14]. The lncRNA *Arid-IR* promotes NF-kB-mediated kidney inflammation by targeting *NLRC5* transcription [15]. The cell cycle changes during aging. Previous studies have shown that lncRNAs are related to cell proliferation and are closely related to cell cycle progression. LncRNA *ANRIL* inhibits vascular smooth muscle cell senescence by alleviating cell cycle arrest [16]. The lncRNA *CASC11* can promote gastric cancer cell apoptosis by accelerating the cell cycle process [17]. The study of lncRNAs in kidney aging is still in its infancy, and we asked whether lncRNAs could regulate the process of kidney aging.

In this study, we aimed to investigate the expression changes of lncRNAs between mouse kidneys of different ages and constructed a lncRNA-mRNA coexpression network to illuminate the role of lncRNAs in kidney aging. Our findings provide new evidence for understanding the potential role of the lncRNA-mRNA coexpression network in the pathogenesis of renal aging.

## Results

Unless otherwise stated, we performed all statistical comparisons using Student’s t test.

### Mouse kidney pathology and SA-β-gal staining

**Fig. 1:**
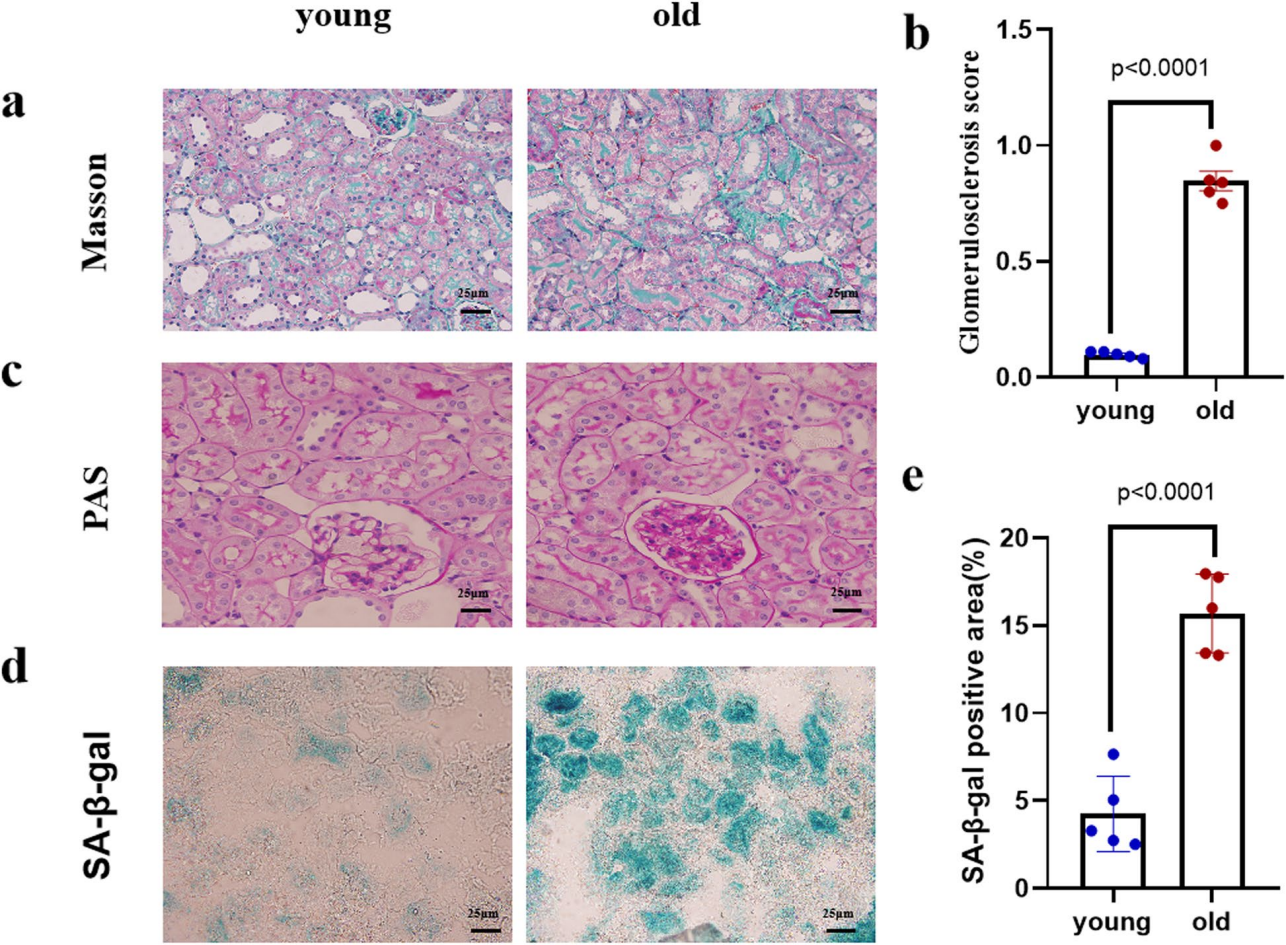
Histopathological changes in the mice kidney. **a**, **c**, **d** PAS, Masson staining and SA-β-gal staining (200×) showed kidney structure changes in mice. *N* = 5; **b** Fibrotic area based on Masson staining were expressed as mean ± SD. *N* = 5; **e** SA-β-gal positive area were expressed as mean ± SD. *N* = 5

Renal histopathology was examined by PAS staining and Masson trichrome staining (Fig. 1). The blue-stained area in the old mouse kidney indicated interstitial fibrosis and collagen deposition (Fig. 1a), and the fibrosis score increased (*P* < 0.05) (Fig. 1b). Old mouse kidneys exhibited thicker glomerular basement membranes, mesangial matrix expansion, and renal interstitial fibrosis by PAS staining (Fig. 1c). SA-β-gal-positive areas in the kidneys of old mice were significantly elevated compared to those in the kidneys of young mice (*P* < 0.05) (Fig. 1d-e). These results indicated that aging leads to the aggravation of renal fibrosis.

### Differential expression of lncRNAs and mRNAs between young and old mouse kidneys

**Fig. 2:**
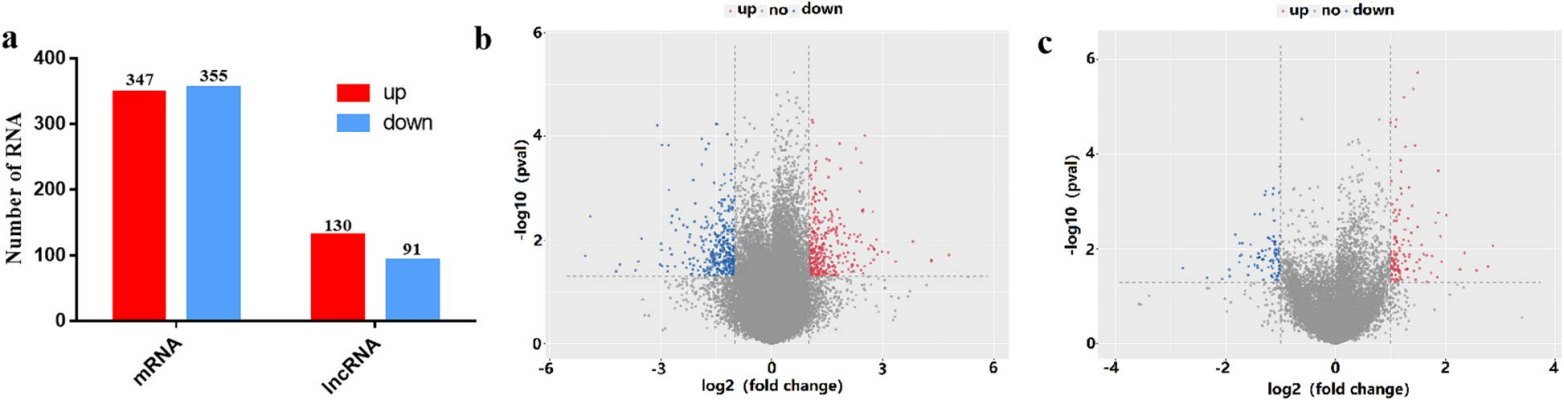
General analysis and volcano map in young and old mouse kidney from RNA-seq. **a** Differentially expressed mRNAs and lncRNAs in young and old mouse kidney detected by RNA-seq; **b** Volcano of mRNAs in young versus old mouse kidney; **c** Volcano of lncRNAs in young versus old mouse kidney. Red indicates up-regulated mRNAs or lncRNAs, blue represents down-regulated mRNAs or lncRNAs, and grey indicates non-regulated mRNAs or lncRNAs

To determine the mechanism of RNAs in aging mice, we analyzed lncRNA and mRNA expression in the kidneys of C57BL/6J mice of different ages by RNA sequencing. A total of 79,320 mRNA transcripts, 10,662 known lncRNA transcripts and 11,412 novel lncRNA transcripts were detected by sequencing. A total of 42 known and 179 novel differentially expressed lncRNAs and 702 differential mRNAs were expressed in the mouse kidney. The histogram and volcano plot showed that there were 347 upregulated mRNAs transcripts and 355 downregulated mRNAs transcripts in the old mouse kidney and 130 upregulated lncRNAs transcripts and 91 downregulated lncRNAs transcripts in the old mouse kidney (Fig. 2).

### Aging-related differentially expressed mRNAs

**Fig. 3:**
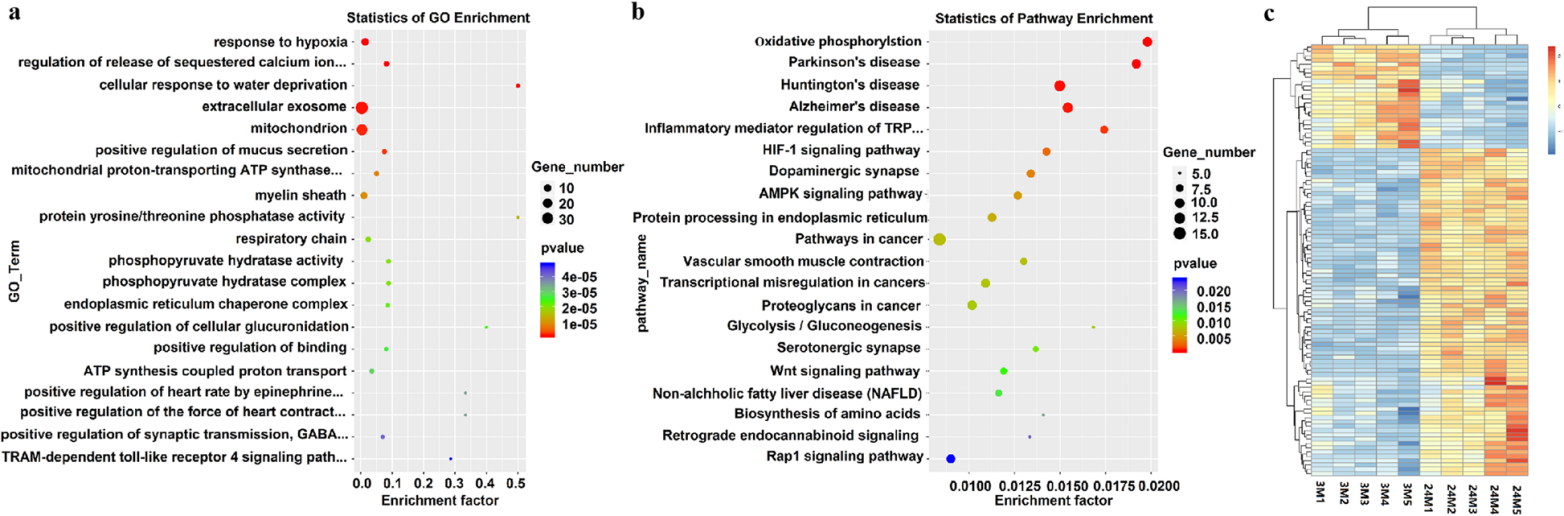
Age-related differentially expressed mRNAs from RNA-seq. **a** GO analysis of mRNAs in young versus old mice kidney; **b** KEGG [18–20] analysis of mRNAs in young versus old mice kidney; **c** Heatmap of mRNAs in young versus old mice kidney. Orange represents an increase in gene expression; blue indicates a decrease in expression levels of gene; white indicating that there is no change in gene expression level. The brightness of the color indicates the degree to which the gene expression level is increased or decreased

To further explore the role of mRNAs in the renal aging process, GO and KEGG analyses were performed on mRNAs differentially expressed in the mouse kidneys (Fig. 3). GO enrichment analysis showed that differentially expressed mRNAs were enriched in biological processes (e.g., phosphorylation, oxidation–reduction process), molecular functions (protein binding, metal ion binding) and cellular components (e.g., membrane, cytoplasm) (Fig. 3a). KEGG pathway enrichment analysis revealed that differentially expressed mRNAs participated in aging-related pathways, such as oxidative phosphorylation, the AMPK signaling pathway, the Wnt signaling pathway, the Rap1 signaling pathway, and age-related disease (Fig. 3b). The differentially expressed mRNAs were included in the heatmap analysis (Fig. 3c).

### mRNA-lncRNA coexpression network and qRT–PCR verification of the lncRNAs

**Fig. 4:**
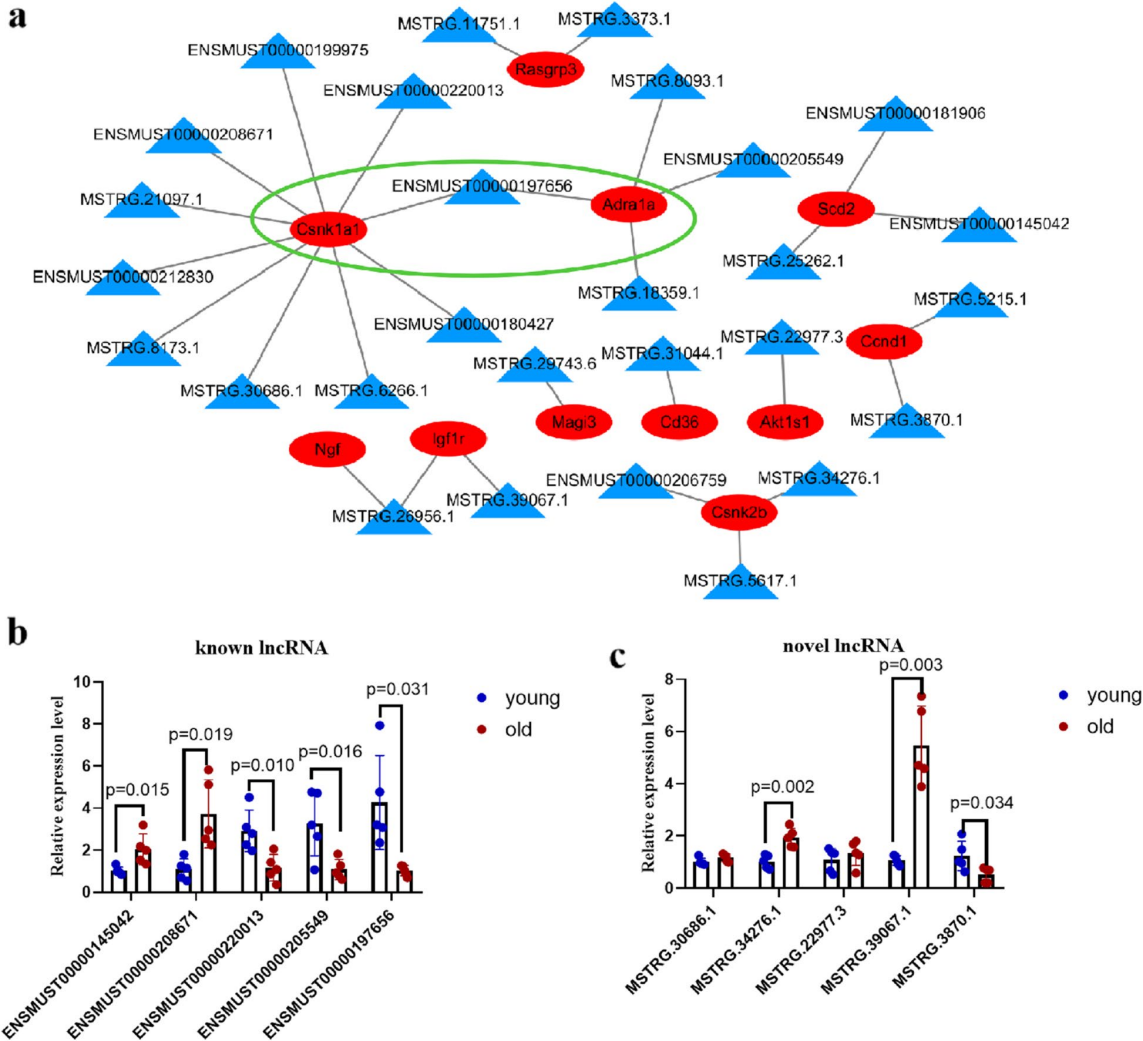
**a** Interaction of coexpressed mRNA-lncRNA. Triangle blue nodes represent lncRNAs; Circular red nodes represent mRNAs. **b** Known lncRNAs relative expression level detected by qRT-PCR. Black indicates young mouse kidney, grey represents old mouse kidney. *N* = 5. **c** Novel lncRNAs relative expression level detected by qRT-PCR. Black indicates young mouse kidney, grey represents old mouse kidney *N* = 5

We observed the potential trans-regulated target genes of lncRNAs using RIsearch analysis. A total 22,917 pairs were obtained between mRNAs and lncRNA transcripts based on trans energy >-70. Then, we choose the pairs with Spearman correlation coefficients > 0.9 or <-0.9 between mRNA-lncRNA based on the RNA expression levels, including a total of 11 mRNAs and 28 lncRNA transcripts (Fig. 4a). Then, 5 known lncRNAs and 5 novel lncRNAs in the mRNA-lncRNA coexpression network were selected to validate the accuracy and reliability of the RNA-seq data. The lncRNAs were described in the heatmap analysis. We designed appropriate primers and amplified lncRNAs. Similar to the RNA-seq data, the qRT–PCR results showed that 5 known lncRNA transcripts, ENSMUST00000145042, ENSMUST00000208671, ENSMUST00000220013, ENSMUST00000205549, and ENSMUST00000197656 (*Gm43360*), and 3 novel lncRNA transcripts, MSTRG.34276.1, MSTRG.39067.1, and MSTRG.3870.1, were consistent with the sequence data (Fig. 4b-c). In addition, the other two lncRNAs were consistent with the sequencing results, but the results were not statistically significant (Fig. 4c). In general, the qRT–PCR results were consistent with RNA-seq data.

### Correlation of lncRNA *Gm43360* and its potential target mRNAs

**Fig. 5:**
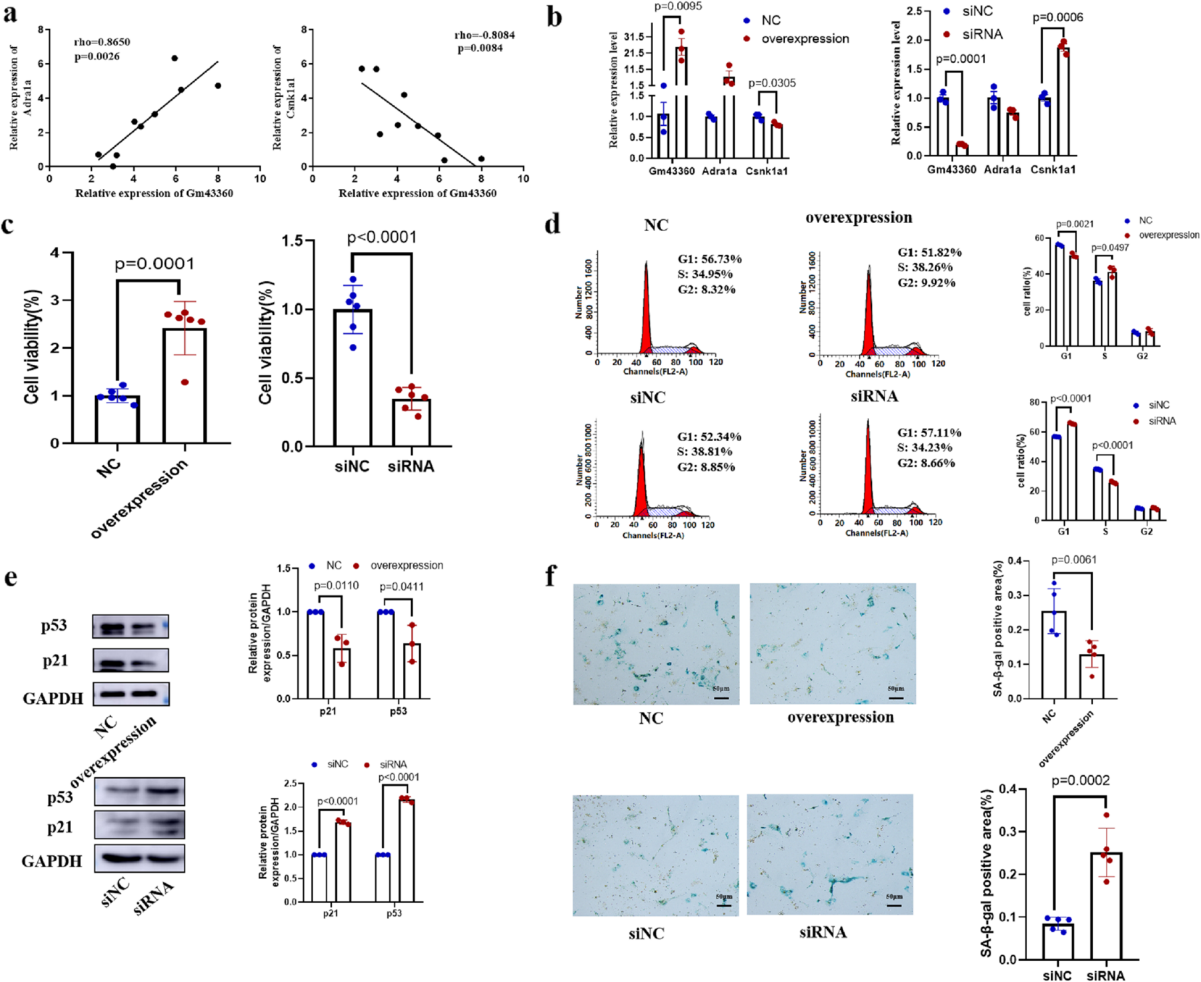
Correlation of lncRNA *Gm43360* and its potential target mRNAs. **a** Spearman’s correlation between lncRNA *Gm43360* expression and Adra1a and the negative correlation between lncRNA *Gm43360* expression and Csnk1a1 in the 9 matched samples from RNA-seq. **b** Adra1a and Csnk1a1 expression level was measured by qRT-PCR. *N* = 3. **c** CCK-8 assays of cell viability. *N* = 6. **d** Cell cycle distribuion were detected by flow cytometry. *N* = 3. **e** The expression of p53 and p21 were measured by Western blotting. *N* = 3. **f** SA-β-gal staining showed that the expression of SA-β-gal were decreased by overexpressed plasmid of *Gm43360*. *N* = 5

LncRNA *Gm43360* was selected for study because it connected two lncRNA-mRNA coexpression networks. The expression level of *Adra1a* was positively correlated (rho = 0.8650, *p* = 0.026) with that of *Gm43360, Csnk1a1* (casein kinase 1A1) was negatively correlated (rho=-0.8084, *p* = 0.0084) (Fig. 5a). To further verify the correlation of lncRNA *Gm43360* and its potential target mRNAs *Adra1a* and *Csnk1a1*, an overexpression plasmid of lncRNA *Gm43360* was designed in this study. The expression of *Csnk1a1* was decreased as expected, and *Adra1a* expression also decreased, but not significantly. Next, we knocked down lncRNA *Gm43360* by siRNA to detect whether the expression of its target mRNAs was affected. As expected, Csnk1a1 was increased in lncRNA *Gm43360* knockdown cells relative to the siRNA negative control group (Fig. 5b). Then, we detected the role of lncRNA *Gm43360* in the cell cycle. The cell counting kit-8 (CCK-8) results showed that lncRNA *Gm43360* promoted cell viability (Fig. 5c). The results showed that lncRNA *Gm43360* increased the percentage of S phase cells and decreased the percentage of G1 phase cells compared with the negative control (Fig. 5d). The p53 and p21 proteins were involved in the regulation of the cell cycle and thus were the marks of cell aging. The expression levels of p53 and p21 were significantly decreased in the overexpression group relative to the control group. The expression levels of p53 and p21 were significantly increased in the siRNA group relative to the siRNA control group (Fig. 5e). Overexpression of lncRNA *Gm43360* decreased the number of SA-β-gal-positive cells, and β-gal-positive cells increased in the group of transfect *Gm43360* (Fig. 5f). Taken together, these results indicated that lncRNA *Gm43360* inhibited the senescence of renal tubular epithelial cells by inhibiting the expression of *Csnk1a1*.

## Discussion

In this study, we evaluated the mRNA expression data from young and old mouse kidneys and conducted bioinformatic analyses that disclosed the functions of the aberrantly expressed mRNAs. We observed 347 upregulated mRNAs and 355 downregulated mRNAs as well as 130 upregulated lncRNAs and 91 downregulated lncRNAs in old mouse kidneys compared with young mouse kidneys. In addition, these mRNAs were shown to be involved in aging-related pathways, such as oxidative phosphorylation, the AMPK signaling pathway, the Wnt signaling pathway, the Rap1 signaling pathway, and age-related disease, which demonstrated the functions of differentially expressed mRNAs in the pathogenesis of renal aging.

The long noncoding RNA *NEAT1* is a protective factor in the progression of kidney fibrosis in renal tubular epithelial cells [21]. Coincidentally, in our sequencing results, lncRNA *NEAT1* was expressed at lower levels in the kidneys of aged mice than in young mice, which is consistent with previous findings. LincRNA-*Gm4419* accelerates inflammation and fibrosis by NF-kB/NLRP3 inflammasome-mediated mechanisms in diabetic nephropathy [22]. In our sequencing results, we also found that the expression of *Gm4419* was higher in the kidneys of aged mice than in young mice, but there was no significant difference. LncRNA *Gm43360* is located on chromosome 5 (Chr5:122494022–122,494,908, 2887 bp). According to the UCSC Genome Browser, *Gm43360* is located in the intron of the protein-coding gene Atp2a2. However, there have been no reports about the involvement of lncRNA *Gm43360* in diseases to date. In this study, knockdown of lncRNA *Gm43360* promoted renal tubular epithelial cell senescence. In addition, we identified many differentially expressed lncRNAs in the sequencing results and investigated them.

LncRNAs were classified into *cis* regulation or *trans* regulation based on lncRNA regulatory mechanisms. *Cis*-acting lncRNAs could influence the expression of neighboring genes by depending on *cis-*acting elements, such as promoters, enhancers, and regulatory sequences, which are distances between lncRNAs and mRNAs of less than 100 kb. *Trans*-acting lncRNAs refer to lncRNAs leaving the site of transfection and operating at distant sites (i.e., the distance between lncRNAs and mRNAs is more than 100 kb) [23]. The lncRNA *MAAT* increases the expression of the neighboring gene *Mbnl1* through a *cis*-regulatory module [24]. The lncRNA *Pnky* plays a role as a *trans*-acting regulator in cortical ddevelopment [25]. In this study, there was a transregulatory relationship between lncRNA *Gm43360* and *Csnk1a1*. *Csnk1a1* is a tumor suppressor gene [26] that encodes a protein that participates in the cell cycle and the cell division process [27]. *Csnk1a1* downregulation induces a senescence-associated inflammatory response with growth arrest in colorectal tumors [26]. *Csnk1a1* was downregulated when *Gm43360* was upregulated; thus, it is likely that lncRNA *Gm43360* participates in kidney aging by regulating *Csnk1a1* expression.

## Conclusions

Our investigation of the lncRNA-mRNA coexpression network in kidney aging revealed a lncRNA, lncRNA *Gm43360*, may play a protective role in kidney aging and expanded our understanding of the mechanisms involved in kidney aging.

## Materials and methods

### Samples

Young (3-month-old) and old (24-month-old) male C57BL/6J mice were purchased from the Experimental Animal Center of Xiamen University and were raised in a standard environment. All mouse had free access to food and water. The mice were acclimated to the new facility for a month before they were sacrificed. The mice were anesthetized with urethane by intraperitoneal injection at a dose of 750 mg/kg before specimen collection. Young and old mice were sacrificed on the same day. Residual mouse kidney tissue was used for sequencing in each assay. RNA-seq and histopathology were performed on separate kidneys from the same mouse. Kidney tissue was collected from mice. One kidney was immediately immersed in 10% neutral buffered formalin for subsequent section embedding, and the other kidney was divided into several tissues and immediately placed in liquid nitrogen and then stored at -80 °C.

 The study was supported by the Ethics Committee of the First Affiliated Hospital of Xi’an Jiaotong University (Shaanxi, China) (No. 2018-G-164). All methods were carried out in accordance with the animal ethics guidelines and regulations. This study was carried out in compliance with the ARRIVE guidelines.

### Kidney histopathology

Renal tissue samples of young and old mice were fixed in 10% paraformaldehyde solution overnight. Then, the sections were dehydrated, paraffin-embedded, and sectioned at 4-µm thickness. PAS and Masson trichromatic staining were performed using standard protocols. Images were captured by camera, and the staining-positive area was measured. The area for the histopathology camera images was calculated with Image-Pro Plus 6.0.

### SA-β-gal staining

SA-β-gal activity was analyzed using an SA-β-gal staining kit (Cell Signaling Technology #9860) according to the manufacturer’s protocol. The area of positive staining was measured, and SA-β-gal-positive cells were calculated using Image-Pro Plus 6.0.

### RNA extraction library construction and sequencing

Samples (each 5 mice in young and old mice) were used for lncRNA and mRNA expression analyses. Young mice were numbered 3M1, 3M2, 3M3, 3M4, and 3M5, and old mice were numbered 24M1, 24M2, 24M3, 24M4, and 24M5. Total RNA was extracted using Trizol reagent (thermofisher, 15,596,018) following the manufacturer’s instruction. The total RNA quantity and purity were analysis of Bioanalyzer 2100 and RNA 6000 Nano LabChip Kit (Agilent, CA, USA, 5067 − 1511), high-quality RNA samples with RIN number > 7.0 were used to construct sequencing library. After extraction of total RNA, mRNA was purified from total RNA (5ug) using Dynabeads Oligo (dT) (Thermo Fisher, CA, USA) with two rounds of purification. Following purification, the mRNA was split into short fragments using divalent cations at high temperature (Magnesium RNA Fragmentation Module (NEB, cat.e6150, USA) under 94℃ 5-7 min). Then the cleaved RNA fragments were reverse-transcribed to obtain the cDNA by SuperScript™ II Reverse Transcriptase (Invitrogen, cat. 1,896,649, USA), which were next used to synthesise U-labeled second-stranded DNAs with E. coli DNA polymerase I (NEB, cat.m0209, USA), RNase H (NEB, cat.m0297, USA) and dUTP Solution (Thermo Fisher, cat.R0133, USA). An A-base was then added to the blunt ends of each strand, preparing them for ligation to the indexed adapters. Each adapter contained a T-base overhang for ligating the adapter to the A-tailed fragmented DNA. Dual-index adapters were ligated to the fragments, and size selection was performed with AMPureXP beads. After the heat-labile UDG enzyme (NEB, cat.m0280, USA) treatment of the U-labeled second-stranded DNAs, the ligated products were amplified with PCR by the following conditions: initial denaturation at 95℃ for 3 min; 8 cycles of denaturation at 98℃ for 15 s, annealing at 60℃ for 15 s, and extension at 72℃ for 30 s; and then final extension at 72℃ for 5 min. The average insert length for the final cDNA librarys were 300 ± 50 bp. Finaly, we performed the 2 × 150 bp paired-end sequencing (PE150) on an Illumina Novaseq™ 6000 (LC-Bio Technology CO., Ltd., Hangzhou, China) according to the manufacturer’s protocol [28].

### Bioinformatics analysis

#### Sequence and filtering of Clean Reads

A cDNA library constructed by technology from the pooled RNA from kidney samples of mice was sequenced run with Illumina NovaseqTM 6000 sequence platform. Using the Illumina paired-end RNA-seq approach, we sequenced the transcriptome, generating a total of millon 2 × 150 bp paired-end reads. Reads obtained from the sequencing machines includes raw reads containing adapters or low quality bases which will affect the following assembly and analysis. Thus, to get high quality clean reads, reads were further filtered by Cutadapt [29] (https://cutadapt.readthedocs.io/en/stable/, version:cutadapt-1.9). The parameters were as follows:

1. removing reads containing adapters;
2. removing reads containing polyA and polyG;
3. removing reads containing more than 5% of unknown nucleotides (N);
4. removing low quality reads containing more than 20% of low quality (*Q*-value ≤ 20) bases.

Then sequence quality was verified using FastQC [30] (http://www.bioinformatics.babraham.ac.uk/projects/fastqc/, 0.11.9). including the Q20, Q30 and GC-content of the clean data.

After that, a total of G bp of cleaned, paired-end reads were produced. The raw sequence data have been submitted to the NCBI Gene Expression Omnibus (GEO) datasets with accession number GSE154223. The reference genome/annotation was Mus_musculus.GRCm38. The source and version of the gene annotation used for analyses was Ensembl_v88.

#### Transcripts assembly

Firstly, Cutadapt [29] was used to remove the reads that contained adaptor contamination, low quality bases and undetermined bases. Then sequense quality was verified using FastQC (http://www.bioinformatics.Babraham.ac.uk/projects/fastqc/). We used Bowtie2 [31] and Hisat2 [32] to map reads to the genome of mouse. The mapped reads of each sample were assembled using StringTie [33]. Then, all transcripts from kidney samples were merged to reconstruct a comprehensive transcriptome using perl scripts. After the final transcriptome was generated, StringTie [33] and edgeR [34] was used to estimate the expression levels of all transcripts.

#### LncRNA identification

First of all, transcripts that overlapped with known mRNAs and transcripts shorter than 200 bp were discarded. Then we utilized CPC [35] and CNCI [36] to predict transcripts with coding potential. All transcripts with CPC score <-1 and CNCI score < 0 were removed. The remaining transcripts were considered as lncRNAs.

#### Differentially expressed analysis genes (DGEs) ananysis

StringTie [33] was used to perform expression level for mRNAs and lncRNAs by calculating FPKM [37]. Genes differential expression analysis was performed by DESeq2 software between two different groups (and by edgeR between two samples) [34, 38]. The genes with the parameter of *q* value below 0.6 and absolute fold change > 2 were considered differentially expressed genes.

#### Target gene prediction and functional analysis of lncRNAs

To explore the function of function of lncRNAs, we predicted the cis-target genes of lncRNAs. LncRNAs may play a *cis* role acting on neighboring target genes. In this study, coding genes in 100,000 upstream and downstream were selected by perl script. Then, we showed functional analysis of the target genes for lncRNAs by using the scripts the BLAST2GO [39]. The full commands link to the public domain was Jie-Li/README.md at main · dandan-li/Jie-Li (github.com).

### Gene ontology (GO) categories and Kyoto Encyclopedia of Genes and Genomes (KEGG) analysis

The bioinformatics analysis for RNA sequencing was performed using OmicStudio tools (http://www.omicsudio.cn/tool). Gene Ontology (GO) functional enrichment analysis and Kyoto Encyclopedia of Genes and Genomes (KEGG) [37–39] enrichment analysis were used to analyze the biological functions of the predicted target genes. GO analysis clarifies the main biological processes through three aspects: cell composition, molecular functions and biological processes (http://www.geneontology.org/). The analysis first puts all differentially expressed genes and background genes in the GO database. Each item is mapped, the number of genes in each item is calculated, and the hypergeometric distribution is used to perform hypothesis testing to obtain the *P* value of the enrichment result. The lower the *P* value, the more significant the enrichment result. KEGG is a database resource that analyzes the differentially expressed mRNAs by genetic biology to explore important pathways related to target genes (https://www.genome.jp/kegg/). The results are expressed by *p* values; the lower the *p* value, the more significant the enrichment result.

### qRT–PCR

GAPDH was used as the endogenous control. Then, the relative expression levels of unknown genes were calculated. All primers were designed and synthesized specifically for this experiment. Primers for GAPDH have been commercialized. The specificity of Adra1a and Csnk1a1 primers was identified by the single peak of the melt curve.

### Bulid mRNA-lncRNA coexpression network

Transregulation was based on RIsearch, and then the trans energy was calculated. The smaller the trans energy was, the higher the possibility of binding. Then, the correlation coefficient of mRNA-lncRNA was also calculated. The mRNA-lncRNA network was constructed by choosing a Spearman correlation coefficient exceeding 0.9. The mRNA-lncRNA coexpression network was constructed using Cytoscape software (v3.7.1).

### Cell culture

Mouse renal proximal tubular epithelial cells (MRPTEpiCs) were cultured in epithelial cell medium-animal (EpiCM-a, Cat. #4131) containing 2% fetal bovine serum (FBS, Cat. #0010) and Epithelial Cell Growth Supplement-animal (EpiCGS-a, Cat. #4182) without antibiotics in a humidified incubator at 37 °C and 5% CO_2_. The culture medium was changed every 2–3 days. The cells in the logarithmic growth phase were subcultured when the growth reached 90%. The cell suspension digested by trypsin was seeded into 6-well plates with 2*10^4^-5*10^4^ cells in each well. The cells used for transfection were all between generations 2 and 5.

## Plasmid construction and cell transfection

The lncRNA *Gm43360* overexpression plasmid was constructed by GeneChem (Shanghai, China). The plasmid backbone was GV658, and the CMV promoter drove lncRNA expression. The cloned nucleotide sequence is provided in the supplementary material. For lncRNA *Gm43360* knockdown, three lncRNA ENSMUST00000197656-target siRNAs (lncRNA *Gm43360*-siRNA1, 5’-CCUUCACUCCAGCUGGUAATT-3’; lncRNA *Gm43360*-siRNA2, 5’-CCCUGUCACUCAUGAAGUUTT-3’; and lncRNA *Gm43360*-siRNA3, 5’-GGUCAAAUAACUCAAUGGGTT-3’) were designed and synthesized at GenePharma (Shanghai, China). According to the manufacturer’s protocol, cells were transfected with 2500 ng of plasmid or 100 pmol of siRNA with 6 µl of Lipofectamine™ 2000 Transfection Reagent (Invitrogen, USA) per well. RNA and protein expression levels were detected at 48 h after transfection, and each experiment was repeated at least three times. Real-time quantitative PCR was used to validate the efficiency of lncRNA *Gm43360* overexpression and knockdown.

### Western blot analysis

Cultured cells were washed with ice-cold PBS, 120 µl of RIPA buffer (Heart, China) was added, and protease inhibitor and PMSF (Heart, China) were added for 30 min on ice. After collecting cells in different microcentrifuge tubes, the protein concentration was quantified. Loading buffer was added to each sample and then boiled for 7 min. Thirty micrograms of sample was separated by 12% SDS–PAGE (Beyotime, China) and electrotransferred to PVDF membranes (Thermo Fisher, USA). Then, the membranes were blocked with 5% milk. The membranes were incubated with specific primary antibodies: anti-p21 (1:1000, Abcam, ab109199), anti-p53 (1:1000, Proteintech, 10442-1-AP) and anti-GAPDH (1:3000, Proteintech, 60004-1-Ig) overnight at 4 °C. After washing with TBST, membranes were incubated with a secondary antibody for 1 h at room temperature. Protein bands were observed using a chemiluminescence kit (Millipore, USA). The expression of GAPDH was used to normalize protein levels.

### Cell proliferation assay

The capacity of cell proliferation was determined by the cell counting kit-8 (CCK-8) assay. Forty-eight hours after transfection, 90 µl of new medium and 10 µl of CCK-8 solution were added to each well (Beyotime, C0042). The cells were incubated for 1–4 h at 37 °C in 5% CO_2_ and measured at 450 nm by a universal microplate reader (Bio-Tek, USA).

### Flow cytometric analysis

Cells were plated in 6-well plates the day before transfection. Forty-eight hours after transfection, the cells were trypsinized and centrifuged at 1000 rpm for 5 min. Then, the cells were fixed in 70% ethanol at 4 °C for at least 4 h. After centrifugation, RNase A and propidium iodide (PI) staining solution were added to the cells, and the cells were then incubated for 30 min at room temperature in the dark. The stained cells were analyzed using a ACEA NovoCyte (Biosciences, USA).

### Statistical analysis

The measurement data in accordance with the normal distribution are presented as the mean ± SD. The difference between two different groups was determined using two-tailed unpaired Student’s t tests, and a *p* value < 0.05 was considered to be statistically significant. All calculations were carried out using GraphPad Prism 8 (GraphPad Software, Inc., USA).

## Supplementary Information

**Additional file 1.**

**Additional file 2.**

**Additional file 3.**

**Additional file 4.**

**Additional file 5.**

**Additional file 6.**

**Additional file 7.**

**Additional file 8.**

**Additional file 9.**

**Additional file 10.**

## Data Availability

The datasets generated and/or analysed during the current study aree available in the GEO repository. The raw sequence data have been submitted to the NCBI Gene Expression Omnibus (GEO) datasets with accession number GSE154223.

## Abbreviations

lncRNAs: Long chain non-coding RNAs
SA-β-gal: Senescence associated β-Galactosidase
GFR: Glomerular filtration rate
CCK-8: cell counting kit-8
PI: propidium iodide
