# Supplementary material for: Predict the role of lncRNA in kidney aging based on RNA sequencing
Source: BMC Genomics. 2022 Apr 2;23:254. doi: 10.1186/s12864-022-08479-8 (PMC8977006; doi:10.1186/s12864-022-08479-8)
Supplement: Supplementary file 7 — Additional file 7. [file 12864_2022_8479_MOESM7_ESM.docx]

**Additional file 7.** Supplementary figure S1-S2.


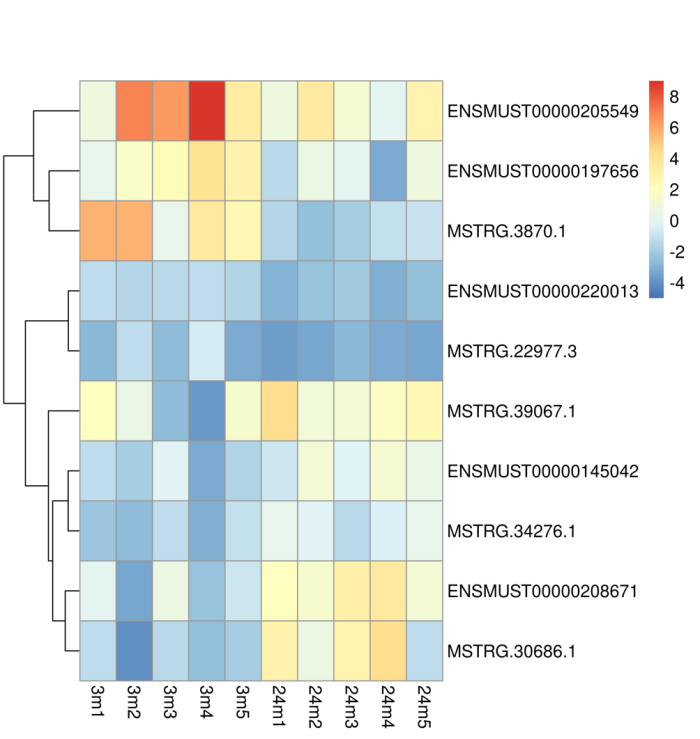


**Additional file 7. Figure S1.** Heatmap of lncRNAs in young versus old mice kidney. Orange represents an increase in gene expression; blue indicates a decrease in expression levels of gene; white indicating that there is no change in gene expression level. The brightness of the color indicates the degree to which the gene expression level is increased or decreased.


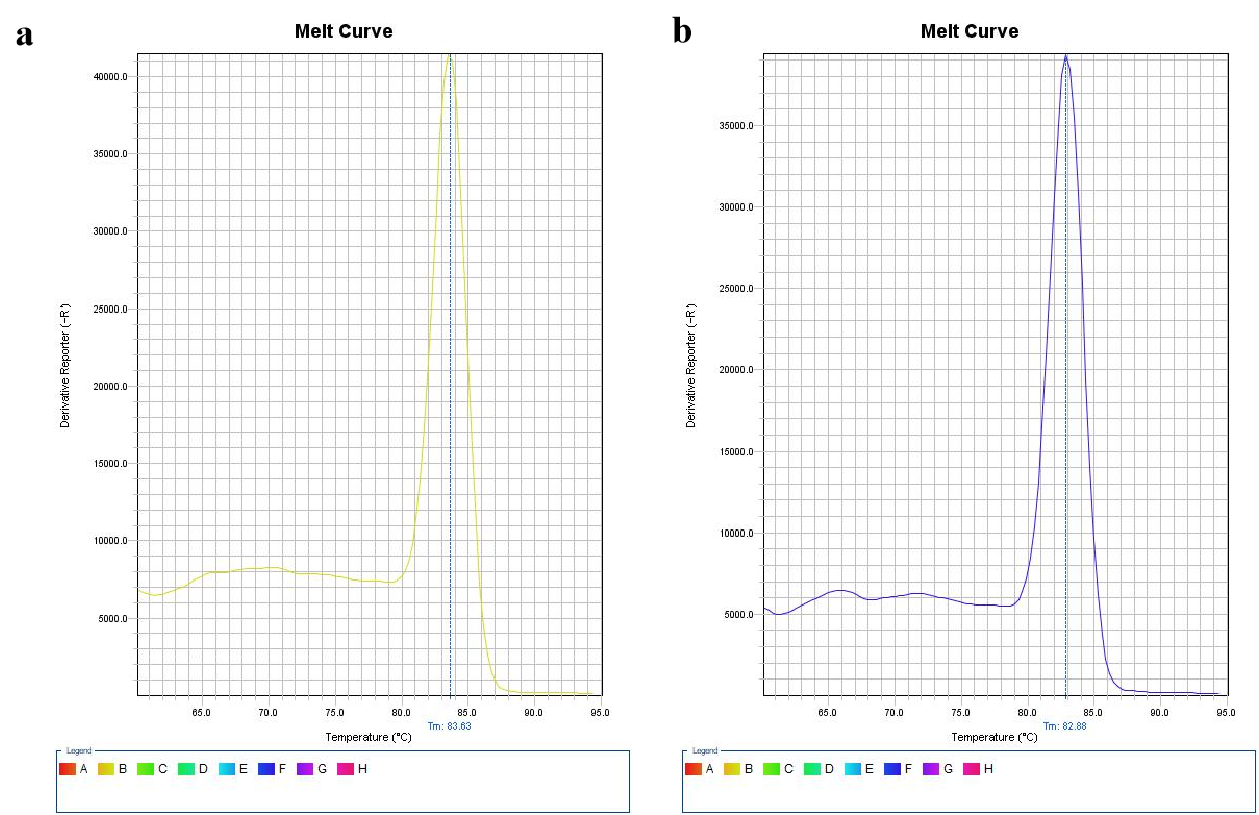


**Additional file 7. Figure S2.** The melt curve of Adra1a (a) and Csnk1a1 (b).
