# Supplementary material for: Predict the role of lncRNA in kidney aging based on RNA sequencing
Source: BMC Genomics. 2022 Apr 2;23:254. doi: 10.1186/s12864-022-08479-8 (PMC8977006; doi:10.1186/s12864-022-08479-8)
Supplement: Supplementary file 9 — Additional file 9. [file 12864_2022_8479_MOESM9_ESM.docx]

**Additional file 9.** The sequence of novel lncRNA.

>MSTRG.30686.1 gene=Alms1-ps2

AGAATGTTGCATGAAAACACTCCTTCCCAAAGATTCATGGCAGAAGTCTTGCTCATGGGGCAGGTGCACT

GGATGATGGAAATAAAACTTGGCTGATCTGAAGACAGAGTGAGCAGTGTTTTCAAATGCAGAATGACATT

CAGATTCTGAAGAAAAAAAGAAAAGAAATTTTTTGAAAGAATTTAGAAATATTTGTATTTCCTTATAAAT

ACTGTTCTGCTTTTTCTTTAGACATAGAACATGTGCTTAACAATCAGACTGGCATGAGTCAAAATATAAG

GATTTGGATAAACTCCAATGCTGTATGACAGATCTGGGACTGGACCCCAGCTCTAAAAGTGCTTGCCTGG

CATGCACAAAGCGCTGGGTTAAATTCCTAGCATGCTCTAAACTGCACTTACAATCCCAGCACTCAGAATA

TTGAGACAGAATGATCAGGATCCAGGTCACCCTCTGCTACACAACCAATTCAAGGCCAGCCTGAACAAAC

TACTTGGTAGCCTATATCAAAAATGAAAAACAAGTGGTCCAAGTACTAGAGAGTACAGAAGCCATTGGAC

ATCTGTGAGTTCAAGGCCACCTTAGTCTACACAGTGAGTTCTAGGACAGCCAAGAATACTTTGTCACAAA

AAAAAATGTATATATGGATTGAACTCTACAATCATGTGACAAAGATGACCTTAAGCACAACCCCTTTAGT

TCTCTACACTATAATTAGTTGCTTTGTAAATCCACCACTTATTCTATTTAGGCTACTTTCTGTATATGTT

CTTTCTTTAAAAAATAGCGGGACAGGTGAGATGGCTCAGTGGGTAGGAGCATCAGACTGCTCTTCCGAAG

GTCCGGAGTTCAAATCCCAGCAACCACACGGTGGCTCACAACCATCTGTAAGGAGACCTGACTCCCTCTT

CTGGAGTGTCTGAAGACAGCTACAGTGTACTTACATATAAATAAATAAATCTTTTTTAAAAAATTGCATC

CTGCTAGTAAGACAGAGATGGGTATTTCCAACTTAAAACAAAAACAAAAAACCTTGAGGTTTCATCATCA

GCATTAAGTGGGTGAAGTAAAAAAAAAAAATTTAAATCCTCACTTTTGAGCAATCATCATTCCGTATCAC

AGTATGTACTGTTGAATACAAGCACATGTTATAGTTTACTAGGCTGACTGTGGTTCTCTGAACCACATTC

AAATTTCATCCCTGATTGCTGGACCATTCAAAGTTATGGCATTCAAAAAACCCTTGTACTGGCTCATGCC

TCTGTTCCTCTTCACAACTGAGCTGCTAAGGTAGAAAACATGTATTTCCCTGTTCTCTTTCCCAAACCAT

TCTCTCCACATTACTACGGAATTATAATAGCTCTATGAGACCAATGGTCTCTTCATAGAGCCAACTCTTA

TCTAGAGTGTGCTCATGCACTAAATTCCCTTTCAAGACACAAATACCAGTCTTCATGGGTTTTTTTAAAT

CACTTCAGTGTCAATATCTACTCTAAGATTTATATTTCAAGCCATGATTTCTCTCAATCAATTCATATCT

ATGTATCATATGCATCACTTTGATGTATAGTCAGTCACCTTAATTTTTAAATGTCTTTAACTGAATTCCA

TTCTAGCCTTTACTAAGAAAGCCATAAAAATAATTAAAACTAATACAAGATCTCAGTAAGGTTTCAAGAT

AAAAATATGTGTTTATAATTTGAATTTTATGTACCTACAGTAAATAAACTGAAACTAAGCATGTCTAGCA

CAAACATAAACATGTAACAAAGGATACCTAGCAAAAATAGCTATGTGTATGTGTTGTGGTGGTTTGAAAG

GGAATGTCCCCTATGAATATTTGTCCCCTATGAATATATAAATATTTGGTTGTTATTGGAGCCACTGTTT

AGAAGGCTTAGAAGGTGTGGCCTTGCTAGATATAGTATATCCCTGGGGACAGGCTTCGAAGTTTCAAGGC

CTTACACCATTCACAGTTCATTCTGTATGCTTCCTCTTTGCACACTTTTAAGATTCACATCATAGTTTGC

TGTTCTCATGGCTTTATCTGCCTTCCCCACCAAGTAGCCACACTTCCCTACCATGATCATGACGGCCTCC

TATCCCTCTGGAACATAAGCCCAAATAAACCCTTCTTTAAGTTGCCTTGGTCGTATTTTATCACAACAAT

AGACAAGAAACTAAAATAAAAGTGGTTTTAACTATCAGTATGACTACTCACTAAATCAAACAGAAAAGCA

ATTTATATAATGTAATACATATATAATATACATAAGTATACACACAGACACACCCTATGCATGTGTGTAT

ATATATACACCATAAAATAAAGTTGAAAGTCCAACACATCCTAGATCAAACTTTACCATGAATCAACAGT

AATCAAGTCAGTTTGGTACTAACAAGAGGATCAAAGCATTAAATCAACCTTAATGGCTGGGCTATTTGGC

TTAGTGTAGCTCCTTACAAAGTTTATCCATGTTATAGCACACTGACAATTTCCTCATGAAGAAGGCATAA

TAACCATTTTTAAATATAAGTCACATTTTTTATTGTTGTTTTGTTTGAGGCAAGGTCTAGTTATGTATTC

TATAATGACCTTGAATTGACTATGTAGATTACAGTGGCCTCTAATTTGTAATCCTCCTGCTCTGCTTTCA

GAGTGCTTGTATTACATGCATATAGCTCTCTATGAAAACTCTTCATTGACCAAGAATTCACTTTTTCCTT

ATCCAATTATCTGATGGACAACTGGATCACTCCTACCTCTTGGTTATAATAAATAACTACAATAAACATG

TGTGATGTTTGAGATCTGGTTTTAAATTCTTTTGAATATATGCCCCAAAGTGGGATTGCTACATTTGTAT

GGTAATTGTTTAATTTTTTTTTTTTTGAGAGAGAGAGAATACATTGTTCTCCAGAACAACTATACCATTT

TACATTTTGCTGAGGTTTGGTCTGTAACTATGTGTTGTAATGCTTAAATTCTGTACCCAAAGGGTTAGGC

CTATGAAGGAATTCTCATGTTTACAAGCCTTTGCTGTGCAAACCTTGTCCCTTAATTTAAAACTATTGGT

TAAATAAAACGGGCTACAGCAAATTACTAGAGGGATTAGAGGTAGGTGGGTTTTGAGTTTCCAGGTGGGG

GTGAAGAGAGGAAGAGAAGAGAAACCCCGAAGAGACAGACAAGAAGGCTCCATGAGAAGTGGTTACCATG

AGCCCATGGCCAGGAAACAGCAAATACTTGGGATACATCACTGGGGAAGGAGCCAGGCCAGCAATTAGAA

AATATTAGGGGTGACCCCCAGTAATTGTCAAAGCCAAATAAAATAACCATAGTCTGAGGCTCATTTATTT

CTAAGCTAGTCGGGGATAAGCTTAAATTGGTTGTACAACGTTCTTGCCGGTGATGCAGGAATTCCTTATT

TTTCCTAACAATTCTTTTGGTAGCTTTTCTACATAATGTGAGCTGTTAGTTCCTTGTGGTTTTGAACTGC

ATTTCTCTTCAATTAGTTGAGTGCATTACGTATTTTCTTGGGTCATGTCTATTTTCTTTGGAGAACTATT

TGTTTAACTCCTTGTACACTTCTAAGTCAGATTTGTTTTTTGTTTTCAAGGCACACATGCTCCTTATAAA

TTCTATGTTTAACTCTCAGCAAATACTTTGGGAAGCCTTGCTTCAGTTTCACTTGTCCATGACCGCTTTT

GCTGTCATGCAAAGAAGTGATTACCAAATACAGTTTCTTGAAGCTTTTCTTTTATGTCTTCTACCAGTTT

TTGGAATTCAGGTCTTGTGTTTATATCTCTAATTCTTCTTGATGTAAGTTTTATGTAATGATTTAACTTT

ATTCTTTTAATGTGGTTATCCAGTTTTTCTATACTCTCAGTTGGAAAGCCTATCCTTTCCTCACTGCATA

GCAGCTATATTCTTATTAAACACTGGTTTTATACATTCTATATGTGAGTAGTTAATGGAAACTTTTCAGG

AAACAGTGTGAGTAGAACTCTCTCTTCTGCTCCTTCCTCTTACATGGAAATTCTTATTTTCTAACAGTTA

AAATTTGACAGCTGAATAATAATGGTTTATGTTTTGTATGTTCTTCTCTTTTATCATAATATTAG

>MSTRG.34276.1 gene=Kat8

GGAGGGGAAAACACAATCCCCAGAAACAGTCTGGCTAGAAGAGAGTTCAGGGCCATCCTGGGCTACAGTC

TCAAAAGACCAGAGAGCCAGGCTTGCTGACCCCTGTAATCCTTGGATTACACTTGGGAAGTAGGCACAGA

AAGATGAGGAGTTCAAGGCCAGCCTTGCCTATGTAGGAAGTTTGAGACTAGCCTGATTACAGGAGACAGT

CTCACAAAAACAAAAAGGGTTACTGCCATGGCTCGGTGGGTACTGGACTTGCCACGTAATCCTAGAGACC

AGAGTTTGATCCCTGGATCCTAATTAAAGGTAGGACTGACTCCACAAAGTTGCTCTATGGTCTCCACATT

CTCAAACCATGGCACACATAACCCCTCCCCCCACATACCATGCATACACACATAGAAGCACAGGAACATA

TCATGCATATACATACATATACTAAATATTTTGAAATATATGTATATATACACACATACACTAATAACAC

ACATACTTTGTTTCTGCTTTTCAAAGATTCCTTATTTTGGATACCTCTGTAGGATGTGTCAGGTAGGGCC

TGGCCCCTAAGACAATGTAAATCCTACCAAGACTTCTAGACCGGCCTGTGCTCCCTCTGGATATTGAGAT

GCTCTGCAATAGCAG

>MSTRG.22977.3 gene=Gm17690

GTCTTTCGGAAGGGAAAGGCGCTCTAGGGCTGCTGCTGTAGTCGGGGCGGAACGGATGTGCTCAGCCCGT

CTCGACACAGTTAACTAAAGCCCCGGATGCTTCCAGACTGTTAGAGGCTCACACGGCTCCCCAAACGCAC

ATTCAAGTGCAAAACCAGGCTTTGTACGTTCTGTGACGCAGAGTTGTGTCCTAAGTGCAGGGCCAGGACT

GCCTTCTGGAGCGACCTATTATTTAGTGAGGTCCACGACGCTCGTGCAGACTCGCCCTCGGCCTAGACTT

GAGCCTGAGGTATTTTGTTTTCCCCTAAGTGTCAAATCTCCGCTTATCTTGGGAAATCTGGGACACGAAA

CCGGGTTGGGTTGCGGGCTGGTATCGAAGCTTTAAGGAGCAGACATGGATCTCTTGAGTTAAAGGCCAGC

CAGGTCTACAGAACGAGTTCCAGGACAGCCATGGCCACACAGAAAACCTGTCTCAAAAAACAGAGTTCCA

GCTGGCCTAATAGACACATAGAGACCATGTCTCAAAAAAGGTGTGCGGACCCCTTCCCCCATATTTTATG

TGTATTTCTTCCAGGCCACGCCTGACTTGGATCCTTTATTTCCTTACAATAATTTGGGTGTTTTTCTCAG

TTCACACCCTCCCCTCCACCCCCACGGCTTTGCTGAGATCTTCATTCAACACAAGAAAATACCAGCAATG

TGTAGAAATACTGTGCTTTAATGAACTCCTTAAATAGAAACGCTGCTATGAAAATATACAGTGGGAGGAG

ACGGTTTCTCCCTTATTCACCTCGTTCCCACAGAAGACTAAGAAAAGATTTCAAAGTAGTGTAGAGAGGT

TGCGAATGAGGTCTCTAGGACATCATTGTTGGGACTGATGAAAAGGGTCTCTCATGTCACAGTTTTGACG

GCCATCCAAAAGGTACAGAATTGCCTGGGAAAAGGGGCAATCACATTCTGAACTGGTCTTGTTTCTTAGT

TTTCAAATCACAAGAAATGAAAATAGCCCCAGAAATCAAACAAATCGGGAGACCTTTAGGTTGGTAGCGC

CACCATCTGCTACAGAAACGGGCCTTCTAAGTGAACTCTGCAGACTGGAATGCCAAAGCTGAGGCGTCAA

GGTCAGACAGGAAGATGAGCAGAGACCCCTGCCCTGCCTCTGAGAACGACGCTAACAAACAGTTTCAGCA

GATCTGAGAGAGGATGGTATTCGGATCTCTTTTCTTGCTCTGGTCCTCGGGAGATATTCTTGGTGTCCAT

TGTCTTAACAGTTCCTGAAGAACAACTGTCTTGCTGAAGGCTGGCTGCAAGTGACAGCTATAAAAGATAG

TGGTGTCTTTCTCTGAGGGACTCGGATATCCTGTCATTCTCATCTAGTAGTGGGAACCTGAGAGAGTCAA

GGGAGTTGAAGTGTAGGTATCTTACCTGCTGTCTAGAGAACCTGCTGTCTGGGCAATGCTCCGGATCGTA

GCCAGGATTAAGGAACTGTGATATCTGTTCCTTGCTGTAGCGTAAATCCCAGTCCCAGACAGACAGATGT

AAGTTAGCAGA

>MSTRG.39067.1 gene=Cdcp1

TCTCCAAAAAAAAAAAAAAAAAAAAGGAGGTGTGGAGCCCAGTATGGGGTTGTAAGTGTCCAGTGTGGTG

AGTCTGACAAAAGATACCTGTTTAAAAGCAACAGTAATCAGGCAGAGAGATGGCTCAGCAGTTAAGAGCA

TGCAGCATTCATTTAGATGACCTGAATTTAATTCCCAGAACCCACATCAGACAGCTTACAACTGCCCAAT

ACCATCTCCAGAGGACATGATACCGTCTCCTGAACTGAAGCACCTGCACTCACATGCACATGCCTGTACA

CACACACATACACACACACACACACACACACACACACA

>MSTRG.3870.1 gene=Rhobtb1

GTGTGTGTGATATGTGATGTGTGTGTGTGTGTGTGTGTGTGTAGTGTGTGTGTGTGTGTAGTGTGTGTGT

ACTTGAAATTCCCAAGCATGAATGGTGTTTTTATTTCTATTTCTTTGGTTAGCTTTTGTTTGGTATTTTG

TTTTTCTTTTGAGGCAGTCTTTTGTAGCCTAGGCTGGCCTCAAACTCATTTTGCAGTTAGAATTGCCTTG

AACTTGTCATTTTCCTGCCTCCACGTCCCTAGTGTACCACCACGCCCAGTTTTATGCAGTGGTGATGACT

GAGCCCAAGGCTTCCAGAATGTTACCCAAGCGTTCTCCCTACACGCCCACCTCTCCAGCCAAAGAGCACA

TTTTGTTTGGTTGGTTGGTTTGCTTTGCTTTGCTTTTGCTTTTACATCACATGTTTTATCCTTAGTTAAA

AATGCACTCAAACATGACAGTTGGCACTGTAGCAAGGCATTGATACTGAATTCTCATGGTGTGGCAAGTT

CTACACACTCTTGTACCATTACAATATTTGGATGTGTTTGCACCGCATTGCAAACTCTTATCTGTCATCC

AGTTCTGGATCAGATCCATTCAATGTGGGCAGACATAATTATGCGGTTTAATTCATTTAAACGGACATAT

TTGCCCCCTCCCCCCATCTTTCTCACGGCAATAGTGGCTGTTCACCATCTTGCTATTACTCCAGATCTTC

CAACAGTTCTCTGCATCATGCAGAGTCAAAGGCTCTGGCCTCCTAGACCACATTTCCCTCTCTCTCTCTC

CTCCCCAATTTTAGTCTGTCCCAGACCCTCTGCGTCTTTTAAGTTCAGCCTGCCTCAGGGCCTTTGCACG

TTCACTTACTGTTCTCCAGTATGACTTGATCGCATATAGCTGTCCTCAGCTTCTAGTCTCTGTTCAAACG

TTGTTACACATACCGCCACCCCCTGCTTTTTTTCCTTCCCAGTTTACTCCTCTACATGACTTATGTCTCC

CTCAGATAAATTTCACATTTAACTCCCTTTTCCTTTTTGGCACATGAACTACGAGAGGGTAGGGATTGCT

GTTGGTTTTACCTACTGGTATCCCAAGTGCTGCACTCATGGACAAATATTAGTTATGTTGGCTTATATCT

TTATTAACTTTTTTTCTCTCTACCTTGAACGCTTTATTTTGCCTTCCTTTCATTTTTTTGGTCTGGTATT

CTACCTTTATCCTAGTTCCATTATGGTAAGACCCAAGTTGCCTAAGTAACTCTAAGCAGAAAGAGTAACT

TAAGAGGTAGGATAAAAGCTGATCTCAAATGGTACCACAGAGCCATAGTGACGGAGGCAGCATAACCCTG

CCATGAAAATGGACATATTGCAAATGGAAAATGGAAAATTTTGCTCCCTGTTAGATTTGGGAAGCAGATG

ACATTTCATTTTTCTTGTCTTTACCCCTATGGAGTTTAACAGGATAAGTCTGCATTTCAGCCACTTTCCT

GGCCCTAATATTTTTGTGATCAGACAAGAACACGGTTAGCACTTAGATAGTAAGTTATCTAGTACAAATC

AGGTTCCCAATGAGGACACTCTTGGGCGAATCATGATGGGGTGGAGGATTGGAAACACCAAGCCTACTGA

TAGTTAATGAAAAGAAAAAAATGAAGATATTTCCTTTATAGCCGAGAACATTTTCCTCCACACATAAAAC

AAATATCGGGCACTTAATGCTGATCCAAGAGGGGGGAAAAAATCTACCTTCCCACAACTGGAAATATAGT

TCATGACAAGGATTTTTTCTTAGTGATGTCATGTGACACACTGGTTAGAATAGCAGTGTCTGAGACATCT

CTGGCCAGCCATTTCTCATTTCTCAGTAGAGGAGATAGAATCTCTGTATCTATGCCTTCCATTAAAAGGC

ATGAGGAGCTGCTAGAGAGATGACTCAGCGGTTAAGAGCATCGACTGCTCTTCCAGAGGTCCTGAGTTCA

AGTCCCAGCAACCACATGGTGACTCACAACCATCCATAATGAGATCTGATGCCCTCTTCTGGTGTGTCTG

AAGACAGCTACAGCGTACTCACATATAATATAGAATTCTTTATATATATATACCATTCCATGTTTTATCA

TTTCGCAGAGGGACCTTGAAAATGTTAAAGAGTGCTGGGTGGATTCCAGGTTTGCTAAAGTCAAGTCTTC

TAAACAAGCCTCCTTTCAGAAAAGGATTCCAGTCTCTGCTGCTCAGTTCTCTCTTGTGCCCTCTAGTGGC

GTTTCTAGGCATAAGGGAAAAAAGTATGACTTTACCTGAAAAACCCACAACATTGTGAGCAGTGCCAGAG

CTATAGGACCTTGATTTGCCTCTCTGCTGCTGACCTTGCCTCTCAGGTTGGCACTGCGGTCTGTTTAGCG

TAAATATGCAAAGACCACCACCTTCGATTGTTATATCAGCCTCCATCTCCATTTGCCACGTGCAATAAAG

CAAAGCCCAGAGCGAGATAATATATCTTCTACCGAATTAAAGAAAACCTGGACCCCTATTCCCTTTGCAA

TATTGTGCCCCCATTAGTGAGAGATCTTACGCTCTTTAAAAAAAAAAAGCCTCTTGGAAATGTCCCAAGG

GATGTGAACAGCTAAGAACTTTGTCTCAAAAATGGAGGAGCGAACCAGTAAAATGTTTTAACCTAGCTGA

TAGCTCCCGAGAAGCTTTGAAGGATTCTGAAGACACCATTAAAAATTAAATAAAAAGCCTCAGCTTGGAT

TTGTGCATGCTTCAATAGGCAAAAAAGTTATATTTAATGCAGAGGGTGTTGAATCTGAATTAGTACAAAT

ATTATTAATTTAATGTTCATACTGCATTTTATGGTTTATAAACTGGTTTGCCATAATTTTATACACTTTC

CTCACAGTGGCACTATAAAATATATCAGCGTTTTATTTTTTCCGCTTGATGGATGAGGCGAGCCTTAATA

AGTGATTCATTTCTTTCCTGTGCATATTGGTTAACTGGTAAATGATTAATTTATGTCTACATGAACTTCA

TCACTCAGAGTATTTATTTAAGTGTGAGTTGGTATTTATTTTGCCTGCAAAGCATTACAGTATCCAATTA

TATATGCATGTTTTAGAGGCAGTAAAGCAGGCACCAAAGGCTAATGCAACATTAAGTCAGATTTTAAATC

CATAGTAAATTCCAAGTTAAGGTATTTAAATCAGCAGCCCCCCTTCCTCGCTGCTCATGAGGGTGAGAAA

TGGCTCCTCTCTAAAACAGCAAAGGGGCACCTGGGCAGTCACCAAGGAAAGACGCATTGAGATTTTTCAG

AGTTCAGAATATTAACTAAAAAAAAAAAAAAAAAAACAACCCAAAACACTGACCCATTACTTTCAATGAG

GACAGAAAAATAATCAAGTTCTTTCCATAAATCTGCCTGCACAACTGTTTCCTGACCGACTGTGGAGCTC

TTGATTTGCCTCCCTTTGGTCTTCTCTTAGTGGGCTTTGTCAACTTTGCACCCTTAAAGTAACTGTTTCC

GTGATCACTGTTTCCTTTCCTACTGTTGTGGCTGGAATCCTCTGGACAAAAGCATCTTATGGAGGAAGAG

CTTGTTTTTGCCTCACAGTTCAAAAATCCCACAAGCGCCGGGGAGTCAAGTCCGCCTACGTGGGAAGCAG

CCAGTCACATTTCATCCAAGCTCAGGAAGGAGAGTGGGGTGACGAATGCGTCTTTCTGCCTTCTCAGTTC

ACACTCTCTATTTAACCAGTTCTGGACGCTAGGCAAGAAACGGTGCCATCATAGACGGGGTCTTCCCATA

ACAACACAGCCATCACCCACAGGCATCCCAGTCCCGCCTCCCAGAGGATTCTAGATTCTGTAGAGCTGAT

TAATGACGTCACACCATGCTTGTATAGAGAAGCCTGGGTAAAACCACATACAAGGATGTGCTGGGGTCCG

CTTTAAAAGACCTGCAAGTTCTTTTGCTTCCTCCTCAGCACTATCTCCAGGGCTGTGAGTTAACATCTTT

CCCAGTGTGGAAGGTGACTAAACTCTAAGATTCCCAAAATGTTTCCTCCCTCAGCTCTGATTCAGCTGCA

GGGGGAGCAAGGTGGGGGGTAGCGAGGGGGCCGCATCACACTGCTTCTGATGCCCCTCTCCTGCCGCTTA

CACCGTGATACCGTGATGTTTCTCTCACGATACCCTCCCTATCATGATAACCAGGAAATGATGTCGCTAC

CATGATAACCCCTATCACGTTGCCCCTACCTGGATACCTCTGTTATGACTCTTCCATCTTGCTGTAAATT

CAATCCAACAATTTAGGATGGAGAAGCAGCAGAAAATTAATTTTATTGAGTCAGCTAGAATATGCTTTCT

TTATGTGCTTTCTGTAACCTCCCTTATCTCCAGACCTCAGAACTCTTTGAGACAATTAGACCTAGACTTA

AAAAATAAATACATAAATTTTTCATTGCAAAAACTCCCTGTCCTGGTGTCTCCTGAAAAAAAAAAAATCT

CAAAAAGTCTAAATATTGACAGGGCTACACGTTGTTGAAGGCAGCTGATGGGAAACACATTGTGTGGGTG

AATACGTCAACTCTTCCAAGACACAGGGATTCTCTCTCGAGTGTAAAATGTACTTTTGGAATCATTAAGT

ACTTCAAATCCAGGAAGTGTTCTGAGGCAATTTTAATGAAAATAGTTTTCTTCTTTAAAACATTTCTGTA

CATGTATAAATACACGTGATGGCCCGTTACCACACGTTTAAGTTTGTGTGCTTTGCCACGTAGACAAAAT

GTTAACAAATTTTTAAACAAGAGACATTAATAGAAAGAAATGTCACTGAATTCTCCTCTGTGGGCTTTTG

CTTAAGGATTAAATATAGCACCAACAAAAGAGATCCAGAGCACAGCTTCCTCACTCAGAATTAGCCGACC

CGGTTTCCTGGATTATTTTTATATAGTAATGACTACACACATATATGGAAAGAGAAAATATAAATACTCC

AGCCATCTTACTTGCTGTCAGTAAAACAGGAAAAGATGTGCATGCAATAAAAATAAATGTCCCGAATATA

AAAAGCATAGAAATCCCATGCTTTTGAAACCCCTTTCATCTGGTTTAGATGTCTGATGAGACACTGTCCC

ACCTCTCCAGATTGCCCAGATAGCCAAGATCTCCCAGCATTCCTTGTGAACCACAGGCTGAGAATTGTTT

ACAGTTCTTGCTACCCCCACCCCCTCCTGTCCATTTATTTGACATGTATTACAGCTTCCAAATTGGAGGT

GTCATTTTCCCCCTTGGGAAGTCCACGTCCTTATCTAGCCTTATGAGAGGGTTGTGAGCTTATATCACTT

TCTAGTACACAGTGAGCTAAGTGGGTGGAGAACCAGATAAGATTTCTTCCGTGTCTGTCTTTTCTATAGT

CCTGTCTCATAACATGAGACACAAGCGGGTATGGTCCCTCTTTCTCAGGTACTGTGCCTGGTCCCTCGGT

TCTAGTACGAGAGAACCCTCCTTCTGGTTCAGTGCCGTCTCTAGATATAATTCAGTTAGCTTTTGACAAA

AGGAAATATTTCAAAGACTTTCTTCTAATTTACCTACTTTTAAGTTTACATAAAAGTATGTGCTATATTT

GGATCATGTTAGCTCACGTCTTTTTCCTTATCTTATGGGCCATCTTGGTTTCTTAGCTTCCTTTGTCTCT

TGATCATGTCAAATATGCACACATGATTTTGGGTGTCTATGTAAATTCTGAGAACCGCCAATGAGAGAAT

ACCGTGGCTCACATCTTTCTAAAACCGACTTAACTCGCCTAATATTTTTATGCGGAAAATGTTGAAAACT

AACAGCAGTCATAAGTGAGAACCCTGAACGCTCTTTGAGGGTGATCCAACCAGGTCACCATGATTTATGG

GAGTGGCACCGGGAGAATGTACAGACCCTCGTTTAGCATCCAGGTACCGAGTGCACCATTTGCACAACCA

TGATCTGGAATTTCCCTTTCAGCTTAACATGTCAGTGTCTCATCCACGTGTAACTTAAAAATCCTCCTGA

CTGAACATTCCTACCCTTCAGAAGTTGGTTTATGGGTTTATTTACCGGTGGATAAGGATAGTATCTTAAT

GTAGAGAGAAGACCATGAGGACAATGCCGGGTGATGGGGAAGGATGATGAATGAGCAAAAGGACCCATGG

GGCATGAAAGAGGAAAAGAGTCCAGGCTAGGGAGGGC
