# Supplementary material for: Predict the role of lncRNA in kidney aging based on RNA sequencing
Source: BMC Genomics. 2022 Apr 2;23:254. doi: 10.1186/s12864-022-08479-8 (PMC8977006; doi:10.1186/s12864-022-08479-8)
Supplement: Supplementary file 10 — Additional file 10. [file 12864_2022_8479_MOESM10_ESM.docx]

**Additional file 10.** The sequence of ENSMUST00000197656 (*Gm43360*).

>ENSMUST00000197656 gene=Gm43360

TTGGCCACTAGTTTCTCACCCAGCAATAATTAGTATATCTGTAGAGCCTTTGAGTGTGGGTAGCCTGTGT

TTTCTATTCAACAGGACACATGGAAGGCAAGGTTACTGTACTGGTTAAATTAGGATTCTTGCATTTGATA

ACTATTAGGCATGAGAACAATGTACATCTCTTGACTGCTAAAGGTTTGTGTTCCTCTAGTAATTCTGACA

CTGCTCACCCTATTCCTTCACTCCAGCTGGTAAGGGACGGTGGCCTAAGTTGTCCTCAGGATTGGCTGCC

AAGAAGGAAATTCATACCCACTTTAACAAGGTGTGAAGCTTATCTTAGAGTCACTAATGCCTCACTGACC

TTTTGGAAAAGGCCCTGTCACTCATGAAGTTGAGCAATTTTTCTTCTTTAAGAGCTAATTAAATTTCTTA

CTCTGATTTGTTCTCCTTGGCAAATGTTTGGAAGGGCTGTATCAGCAGCTGTGATCCTGCACCTATCAAA

ATTTATAACTTCCTAAAGAAGTTTTCATTTTGGCGGAGATCCTGGCCTTAATACTAGGAATGCCCTGTTG

AAAGCTTGCAGTCGTGTTTATTACATTCATTCTGGTTTCCTCTTGCCCTACTTGTCAATGGTAATTGACA

TGTGTCAGAGCCTGTATACTTTTTCTTTGATAGGATTCTTTGCTAGTTCAATGTTATTCCTCCTTAGAGT

TTTAATTACCTTTTGGTAACTTGTTATTTGTGGGTTTTTTGTTTTGTTTTTTAAAAAACCTTAGAGAATA

AATGCTATGTGGGCTCATTATTTTACCTTTATTTTACTTATAACATGGGATGTGTGTGTGTGTGTTTACA

CACACATTTTACTTATAACACGGTGTGTGTGTGTGTGTGTGTGTGTGTGTGTGTGTGTGTGTGTGTTTAC

ACACACGTTTGTTTGGAGACAGGGTCTCTTTATTATGTCCTGGAACTCACCTTATAGACCAGGCTGGCTT

CGAACTCGGATCCACTGGCCATTGCCTCCTAAGATCCAGGAGTAAATGGCTTTTGGGGGTGGTGGGGAGG

GGTGGTATATACAAAGGTCAAATAACTCAATGGGATTGGTTCTTTCCTCCCACTTTTATGTGGGTTCCAG

GGATTGAATCAAAGCTTGCCAAGCTTGTAGAGTAAGTGCCTTTACCTGATCAGTCATCTTGCTAGCTCCT

AGGTAATCTTTTAAAGAAAAGGTCTTGGTTGGTGAGATGGCTCAGCGGTTAAGAGCACTGACTGCTCTTC

CAAAGGTCCTGAGTTCAAATCCATCCCAGCAATCACATGGTGGCTCACAACCATTTGTAATGAGATCTGA

CACCCTCTTCAGATAGTAGTGTCTGAAGACAGCTACAGTGTACTTACATATAATAAATAAATCTTTTTTA

AAAAGAAAAGGTCTTTATTATGAAAATGTTTTCATTTTAAGCCATATAGCCTCTCGTCTCACGTGCCCCC

CCCCCCATGTAAAGCAAGGTCCACCTCCCAGATTTAGTCATTCAGGTTGGCAGGTTTTTGATAAAATTGC

CACTGACATTTATCAGGACTTGGCCCTGGAAAGTGGGAGGGAGCTTATGTGCATATGTGACATGGGCATT

GCTGTATGGAGGTCAGAGGACAACTTTGAGAAGTCGGTTATCAGATCCTTGCATTGTGAGTGTGAAACTT

GAACCCAGATCATCAGACTTGGATAGCAAGTGCCTTTACCCACTCATCCATGGCACTTGCCCTCCTAGTT

AGCTTTGAATTCAGATCAGCAAGTCCAACCTATGCTCATGGCACCTTTGGGGGCGTTTAATGATCCTTTT

TCAGGGGTCATTAAGATCACCAGAAAACACAGGTATTTACATTACAATTCATAACAAAGCAAAATTACAG

TCATGAAGTAGCAATGACAATAATCTTATGGTTGGTGGTCACCACATGAGGAACTATATTAAAGGGTCAC

GGCACTAGGAAGATTGGAGTTTTGTTTAAAAAAAAAAAGTGGTGTGTGTGTAGCATTCATATACCTTTTT

GGAATAGTTTGCCTGTTACCGTGTGATTGAATTTCTAGACTGATAGTCTAGGCAGCAGACACTTTTACAT

AATATGCCGCTTCCCCAGCCCAGTTTATTTTATTTATTTGGAGGGTAATTTATATAATTTCTGAAGTGCT

GTATTGCAATACTAGGTGCTGTGGTAAATTGCTTTAAAAAATACATTTCTCGTCTCAGAACAAGCTTGTG

ATTCTGATGTGACCCTCACTAACCCTGAGAAGGTAGAAATTAGGTTCTACTTTGAATTTGAATAAATGGT

CAATTTTTTTTCTCCAAATGTTTGCTTGTTTGGGTGTTTTTTGTTTTGTTTTGTTTTGTTTTTTGAGATG

GGGTTTTTCAGTTTGCAGACCAGGCTGGCCTCTGCCTCCCAAGTGCTGGGATTAAAGGTGTGCACCATCA

CGCCTGGCTACTTTTTTGTTTGTCTTTTAAACTGTAAATTCATGCCATGTATGTCTGTGAATTGATGTAA

ATTTTTAAGAATTTTTTTATTAATTGGGTGTTTGCCTACATGTTTATTTGTATACCACATGTGTTCAGAG

CTTTGGAGGCCAGAAGGGGTCTTTAAGACCATACCTACTCCCCCTCTCTACCCTCTACTACCTGAGTTAC

AGACAACTGCTGGGGATGAAACCTGGTCCTCTGGATGAGCAGCCAGGGTTCTTAACCTGAGTCATCTCTC

CAGGCCCTGGTGTGAACATTAACTAAATCAGTGTGGGTATAGGTGTTTACCTGGTCACCTTTTACTATAA

TAAAATCGGGAATTTTAAGTTTCATTTATTTGAAATGTTACCTATGAAGTGAATGAGATTCTTTCTTTCC

ACCAAAACAAACAAACG
